# Teaching under lockdown: the change in the social practice of teaching

**DOI:** 10.1007/s10734-022-00863-3

**Published:** 2022-05-07

**Authors:** Helena Kovacs, Jessica Dehler Zufferey, Roland Tormey, Patrick Jermann

**Affiliations:** EPFL, Lausanne, Switzerland

**Keywords:** Pedagogical change, Social practice, Lockdown, Emotions, Higher education

## Abstract

Due to the unprecedented situation caused by a global pandemic, the traditional way of teaching that is reliant on face-to-face interaction between teachers and students has been dismantled. This article looks into university teachers’ experiences of teaching under lockdown, with an intention to understand what the change meant in terms of social practice. The research follows a qualitative design, in which ten university teachers were interviewed using a semi-structured interview guide. Three themes interwoven with a common thread were identified through teachers’ reflections, including displacement, routine, and role. The common thread was identified as the interaction between teachers and students, and analysing the quality of this interaction led to understanding the social kernel of teaching as embedded in social practice, suggesting that physical dislocation demands teachers to recreate meaning in the new situation. This change has been seen as difficult, yet unpacking teachers’ perceptions provided valuable lessons for the future.

## Introduction

At the outset of the COVID-19 pandemic caused by the novel coronavirus (SARS-CoV-2), countries around the world went into partial or complete lockdown, leaving only the essential operations running. Most countries announced immediate closures of non-essential services and facilities, which included the closure of all education providers, shifting to home-office mode, and directing teachers, students, researchers, administrators, and school leaders to engage with distant operational methods.

Situated in an engineering higher education institution, the current research tapped into the condition of forced change to teachers’ practice required by the total lockdown. While intellectually challenging, teaching is conceived as a social practice (D’eon et al., 2000; Mardahl-Hansen, 2019) constituted by exercising social agency around things of value and situated in a setting that constructs and enables it (Haslanger, 2018; D’eon et al., 2000). The change caused by the lockdown was the most evidently displayed through the shift from on-site synchronous physical lecturing in auditoriums, classes, and laboratories, to teaching and learning online, often asynchronously and in one’s own private space. It was followed by less visible changes, including personal concerns, sizable alterations in daily routines, elevated overall stress and anxiety, and in some cases dealing with the disease itself (Clabaugh et al., 2021). These changes arguably had an impact on how teaching is conducted, what it constitutes, and how learning emerges without diversity of direct on-site interactions that support student holistic learning. With this in mind, we have developed this paper to understand the meaning of changes imposed by the lockdown with regards to how they impacted teaching from a perspective of social practice.

Our standing premise suggests that teaching is a flexible and dynamic professional practice that is set in a specific socio-cultural context (Mårtensson et al., 2011; D’eon et al., 2000; Mardahl-Hansen, 2019) and continuously exposed to change by internal and external influences (Palmer & Collins, 2006; Trowler & Cooper, 2002). The change is motivated by a drive to achieve educational goals and often by a desire to better student learning, as well as to advance the teaching profession (Hutchings & Shulman, 1999; Trigwell et al., 2000). Following this premise, we observed the lockdown situation as a dramatic external event that impacted a somewhat well-established pedagogical practice. Acknowledging the fact that this practice is highly contextual, two leading qualitative research questions were developed:

1. How did teachers experience the abrupt forced change to their teaching practice?
2. In what ways did the lockdown impact the meaning of teaching as a social practice?

The first question explores the immediate response of teachers to the change in their teaching routines and describes their challenges and reactions. In the non-emergency setting, change can often be difficult or even unwelcomed (Brownell & Tanner, 2012; Everley & Smith, 1996; Hodges, 2006; Le Fevre, 2014); hence, exploring teachers’ experiences during a crisis that made the change inevitable can give valuable insight into the diversity of factors and how we can potentially use them to stimulate a smoother adaptation to novel practices. Through the second question, we dissect the ideas around meaning tied to how teachers saw their teaching to understand the relational aspect of the social practice of teaching.

The article is structured to first provide a theoretical insight into what we consider teaching practice and change, mainly looking into literature on social and relational dimensions of teaching. This is followed by methodological reasoning for the study and information on data and analysis. The sections after provide an illustration of our data grouped around analytical themes, and a discussion that reflects on these results and their relevance.

## Social practices and the context of change

In this section, we explore the literature for developing an understanding of pandemic as a new context in which teaching and learning happened, alongside discussing the change in education, particularly from the pedagogical perspective. From here we dive further into the nature of teaching and explore teaching as a social practice that rests on socially constructed rules, and that is purposed through the interaction between teacher and student.

### Change and the pandemic as a new social educational context

Change is an integral part of social practices; hence we see it in teaching as well. Deliberate pedagogical change is often seen through the lens of innovation, with an understanding that it is an enhanced development across time, which when implemented in the curriculum requires parameters of creativity and overcoming the routine (Badran, 2007; Cropley, 2015). Oftentimes, innovation in education is linked with technology aimed at improving teaching practice and learning outcomes (Hernandez-de-Menendez & Morales-Menendez, 2019). However, pedagogical change has been known as a notoriously difficult and slow process, associated with both political institutionalisation and attitudinal change that often counters faculties’ primary role in the research (Everley & Smith, 1996; Resnick et al., 2010). In addition to this, change in teaching practice often leads to short-term loss of effectiveness that has been established through years of routine-building, and this causes teachers to feel vulnerable, uncertain, and anxious (Hammerness et al., 2007; James, 2010; Trigwell, 2012). Bransford et al. (2007) argue that both innovation and efficiency are important for establishing an optimal adaptability corridor that allows teaching professionals to engage with change in an adequate way. The authors note,

In contrast [to routine experts], adaptive experts are much more likely to change their core competences and continually expand the breadth and depth of their expertise. This reconstructing of core ideas, beliefs, and competences may reduce their efficiency in the short run but make them more flexible in the long run. (2007, 49)

However, it is also quite important to understand that for most institutions and faculty members, change has been a voluntary undertaking, even when taken up due to external pressures such as competition with peer institutions (Everley & Smith, 1996). In the context of forced lockdown due to a pandemic, the pedagogical change lost its voluntary character. The rapid change that the pandemic brought caused great disruption not only by the fact that it dislocated the place of education but also by how education is conducted. From a teaching perspective, this change arguably meant that the mutually understood behaviours and patterns of action that are contextually tied to auditoriums, classrooms and laboratories, shifted to personal spaces in private homes. Adjusting to the change meant adjusting to teaching from home, to digital tools, and to finding appropriate pedagogical solutions for online teaching.

In the recent academic papers on the topic of teaching under lockdown, teachers, especially those without previous online teaching experience, reported spending more time in preparation and seeking out support (Farnell et al., 2021). On the other end, Tartavulea et al. (2020) note that some of the initial technical hurdles were quickly overcome and that institutional support played a critical role in this. Yet, some outcomes show that most teachers would have preferred to return to their traditional methods as soon as possible (Tartavulea et al., 2020). In a study done by Damşa et al., (2021) on teachers’ conduct during the crisis, the authors note that teachers used a rather limited number and types of teaching methods while responding to the emergency online teaching, which occurred mainly due to the lack of pedagogical capabilities and the time necessary to adjust, especially for those who had no or little previous experience with online teaching methods.

Combining what we know about online teaching and teacher-student interaction, a study by Welch & Napoleon (2015) concludes that teachers who embrace a more mindful teaching approach with closer interaction with students are potentially better placed to produce a positive significant impact through online teaching. Garrison et al. (2003) additionally note that distance education needs to combine social, cognitive and teaching presence, and the objective of teaching presence is to design and facilitate the other two for the purpose of achieving meaningful, higher-order learning. This leads to an understanding that the disposition towards the techno-pedagogical aspect of online teaching is closely connected to how teachers view the value of teaching in relation to students’ learning.

### Teaching as a social practice

In this paper, social practice is defined as a contextually formed social behaviour through which specific goals are achieved and specific values are enacted by interpreting shared cultural meanings (Haslanger, 2018). Haslanger points out that in education, for instance:

an academic lecture coordinates a community in producing and distributing knowledge through the interpretation of individuals as professors engaged in knowledge production and as having a responsibility to share the knowledge, of spaces as lecture halls where students […] can gather to learn, of podiums as places where professors stand, and chairs as places to sit quietly for 50 minutes to listen (etc.), all of which is governed by social meanings internalized through participation in academic life (Haslanger, 2018, 245).

From this, we can argue that the contextual setting provides meaning to certain behaviours and that behaviours of certain actors are, thus, taken by mutually understood and agreed on rules. The situatedness of various social practices is defined by space and time, as well as the actors with whom the interactions are made and the institutional structure which defines the frames of a practice (Burr, 2007; Penuel et al., 2016). By understanding the perspective of social practice, the nature of teaching is wrapped around several essential features strongly anchored in its context (D’eon et al., 2000). Firstly, teaching is a purposive practice which means it is defined by and stems from its purpose, and does not hold a meaning without its goal (D’eon et al., 2000; Mardahl-Hansen, 2019). In other words, without students and without a goal to learn and be taught, teaching has little if any purpose or meaning. Closely connected to this, teaching is a rational activity justified by its purpose, which means there is a reason behind a certain activity, action or behaviour that is embedded in its purpose (D’eon et al., 2000). Teachers will think and assess what and how to teach depending on what they see as the ultimate goal, and by doing so, employ a wide range of activities accompanied with a strong moral aspect. In this sense, teaching is more than just projecting a voice in front of student audience; it demands judgement and logic based on highly situated circumstances and the decisions taken need to be in line with a moral standing towards students and society (D’eon et al., 2000). Finally, teaching is a communal practice often hidden behind a single teacher. By this, both the purpose and the act of teaching are moulded by values, beliefs, and norms of institutions, communities, and epistemologies surrounding it. And while there is a set of rules, norms, principles, and standards that are accompanied by teaching, these often remain implicit to the given educational context (e.g. institution) (D’eon et al., 2000; Mardahl-Hansen, 2019).

By agreeing with Priestley et al. (2015, 4) in defining teaching ‘as a complex interactive process of communication, interpretation, and joint meaning-making where teacher judgement and decision-making are crucial’, we view teaching as inseparable from its social environment that is strongly tied by relationships among the actors (students, colleagues, leadership) and to physical spaces. Beyond this, as a social practice, teaching is inevitably susceptible to continuous change, making it a dynamic and fluent activity reliant on the context and its relationships.

### Teacher-student relationship as a vehicle of social practice

Drawing on the notions from the previous section, teaching is a purposeful social practice in service of educational goals, most frequently formulated as student learning. However, ‘learning is neither caused by nor an exclusive result of teaching’ (Mardahl-Hansen, 2019, 5); hence, the changes teaching aspires to gain are the changes in how students relate to the social world. This makes both teachers and students agents in educational situations, as they participate from their respective positions regulated by practices, traditions, and norms (Mardahl-Hansen, 2019) often determined in years before reaching higher education and often creating an empathy gap reflected in lack of understanding for the other (Hattie & Yates, 2014). In terms of distance education, there are somewhat contrasting ideas on the relationship between teachers and students. For instance, Saba notes that most distance education relies on the centrality and independence of the learning which is guided by didactic conversations with teachers through using telecommunication modes. On the other hand, Garrison et al. point that, in contrast to the individual independent learning approach, in distant education, ‘higher-order learning outcomes are best enabled in a community of inquiry composed of students and teachers’ (2003, 115).

Literature on the teacher-student relationship seems to suggest that the development of a caring teaching approach combined with a sense of immediacy provides better student learning outcomes (Andersen, 1979; Trigwell, 2012; Walker & Gleaves, 2016; Wubbels & Brekelmans, 2005). More specifically, physical and psychological proximity often demonstrated through non-verbal behaviours (Andersen, 1979; Wubbels & Brekelmans, 2005) can result in higher teaching effectiveness, which is defined by Andersen (1979) as a teacher’s ability to produce an effective behavioural and cognitive learning environment. The literature also shows that there is a relationship between teachers’ emotions and their teaching approach, and it is suggested by Trigwell (2012) that teachers who experience positive emotions regarding their teaching context are more likely to adopt more conceptual pedagogical change and a student-focused approach to teaching. This means that ‘the teachers who describe higher levels of emotion such as pride and motivation and lower frustration are those who also describe their teaching in terms of focusing more on what the student is doing and experiencing’ (Trigwell, 2012, 617). Furthermore, successful interaction with students reflects positively on teachers, as their efforts to reach students are rewarded (Hagenauer & Volet, 2014). On the other hand, negative emotions such as anxiety and embarrassment are positively associated with the adoption of approaches leaning towards information transmission, which are also perceived as ‘safer’ approaches. Negative emotions, as Hagenauer and Volet (2014) note, can come from unfulfilled expectations from students and realising that teaching is only a partly controllable activity. And, indeed, looking from the perspective of social theory of learning (Wenger, 2009), learning develops along the lines of knowledge, meaning, identity and community, and as such emerges through more than just teachers’ input in the classrooms and laboratories. Notwithstanding the fact that teachers do impact the sense of becoming and belonging among students, these aspects together with the other two are also heavily influenced by interactions with peers, coaches, librarians, and through engagement in student associations and extra-curricular academic life, which are often not evident to teachers when they think of student learning.

Combining the notions around the teacher-student relationship, the nature of teaching, and the change driven by the pandemic, it can be argued that teaching under new circumstances had an impact on how social practice is defined and understood within the higher education context. Next to this, it is necessary to acknowledge the important relational and emotional element that teaching practice involves, especially in this case of crisis.

## Methodology and context of the study

This research is positioned within the interpretivist theoretical perspective which allows for exploring data through subjective realities (Cresswell & Poth, 2018; Koro-Ljungberg & Douglas, 2008) that help describe dimensions and complexity of teaching experiences under the situation of a forced change. With this in mind, our principal tool for data collection was a semi-structured interview which we used with a selection of teachers that volunteered to participate in the study.

### Epistemological and methodological framework

Our qualitative research approach was characteristically open-ended and inductive, offering an exploratory departure instead of a finite research expectation (Baker & Edwards, 2012; Cresswell & Poth, 2018), which methodologically was the most appropriate way to explore the lived experiences, the connections between the setting and behaviour, as well as the community and the context. As pointed out by Koro-Ljungberg and Douglas,

[q]ualitative research approaches enable researchers to investigate individuals’ behaviours, associated cultural phenomena, and socio-political influences and processes, in-depth and from the perspectives of the study participants. It allows participants to define factors and highlight influences that they find meaningful and essential to describe their life experiences (2008, 165).

In the current research, we take a social constructivist interpretative framework (Cresswell & Poth, 2018) which linearly connects to the aims of studying the complexity of the contextual change through exploration of realities that are co-constructed between the researcher and research participants.

### Semi-structured interview guide

In alignment with the research questions, we selected semi-structured online interviews which offer the space for participants to tell their stories in a way that is valuable for the meaning-making (Seidman, 2006). The semi-structured interview guide was developed based on previous literature reviews of a range of factors that influence teachers and their practice including, among others, teachers’ understanding of learning and students, teachers’ positioning regarding pedagogical innovation and professional development, emotional aspects of pedagogical change, work-based learning aspects, and communities of practice, as well as institutional features such as organisational learning. Additionally, we were interested in understanding the initial reactions and comparisons between in-person and emergency online teaching.

### Data collection

Conducted in a semi-structured fashion, the interviews consisted of five main question categories, namely (1) introduction and initial reaction to lockdown, (2) development of a routine, (3) reflection on students and their learning, (4) comparisons and challenges, and (5) closure with comments on impacts, lasting changes, and future. Out of a total of 10 interviews, one was done asynchronously through an email exchange, while the rest were conducted synchronously by the lead researcher using Zoom. Using Zoom as a tool in collecting data had also allowed us to understand the materiality of teaching situations and look into the emerging social practice of teaching from isolation. Except this, we decided to include the email interview because asynchronous communication was also part of the emerging practice, and we anticipated that the given answers will provide value to the study.

The interviews took place between April and mid-May 2020, and the interviewing time was on average 40 min. Following Glasser and Strauss (Cresswell & Poth, 2018), interview summaries, memos, and initial analytical notes were taken after each interview throughout the data collection process. It is important to mention that all interviews conducted through teleconferencing tools were video calls which allowed a slightly better conversational setting and interviewing interaction. We note this, as experiences of non-video distance interviewing indicate that the interviews end up being comparatively shorter, more dominated by the researcher, and reduced in themes coverage (Irvine, 2011). Therefore, video calls and adequate conversational positioning were preferred with a deliberate intention to recreate an environment that cultivates a rich exchange of interviewees’ opinions.

### Participants

The participants were all higher education teachers at one of the highly regarded engineering universities in Europe. Our choice of the institution was driven by convenience, and following one of the most common approaches in qualitative studies, we used purposeful sampling to find a selection of participants that fit the needs of the research questions and objectives (Cresswell & Poth, 2018; Merriam & Tisdell, 2016). It was important for us to have participants with a range of backgrounds in teaching, in terms of the number of years of experience, type of teaching (i.e. labs, large classroom), and their familiarity with online pedagogies. While gender did not particularly play a role in the methodological and analytical setup, we did seek to include perspectives of participants identifying as male and female.

This said, at the moment of interviewing, 5 out of 10 participants were involved in teaching large audiences, usually in auditoriums and with first-year students. Among the interviewees, 2 were involved in teaching lab exercises, 3 had prior online teaching experience, and 1 was in a position of leadership. With regard to the duration of their teaching experience, 6 interviewees were considered experienced lecturers with more than 15 years of teaching, 2 were considered mid-career, with between 5 and 10 years of experience, and two were at an early stage in their careers with less than 5 years of teaching experience. Concerning specific subjects, 4 participants came from basic sciences (Mathematics, Physics, Chemistry), 5 from engineering sciences, and 1 from computer and communication sciences. Finally, 7 of the interviewees identified as male, and 3 as female.

### Data analysis procedures

In preparing data for analysis, recorded interviews were transcribed by the lead researcher due to the potential vulnerability of information and ethics procedures for data handling. Transcripts were pseudonymised to protect interviewees’ identities.

There were two main qualitative coding phases done using NVivo software. In the first phase, we engaged with data through inductive open coding which resulted in producing a comprehensive codebook. This was followed by deductive coding, which tested the usability of the comprehensive codebook, resulting in a final codebook with refined code definitions. The final codebook was used in the second round of coding and axial coding, after which the codes were grouped around five categories. These categories helped in developing themes that emerged through considering how the categories interact with each other and with the temporal descriptions noted in the participants’ interviews. In the result section, we explore all three categories and the intertwined element of interaction.

### Trustworthiness

To achieve trustworthiness in the procedures and results, we conducted two steps of interrater agreement, accompanied by three internal validity meetings, one peer group validation session, and a participant validation (‘interviewee check’) session (Cresswell & Poth, 2018). We approached data analysis iteratively and transparently throughout the period of interpretation and analysis of data, thus investing efforts to ensure the quality of both making and handling data (Walther et al., 2013).

To begin with, we sought to establish a trustworthy grasp of data through inductive and deductive cross-coding exercises. The intercoder agreement ensured the validity of the final codebook, coding patterns, and further coding procedures undertaken by the lead coder. After the initial analysis of all data, another round of internal validity around analytical steps was performed through re-examining the initial conclusions. This was followed by an external peer group validation, which gathered peer researchers from other groups and labs, and teaching advisors. During the peer validation, procedures, analysis and conclusions were exposed, opened to questioning and scrutiny, and feedback was taken into consideration in developing conclusions.

In the last step, a participant validation session was initiated through an online meeting with three interviewees. The session exposed the main conclusions, alongside basic analytical and methodological procedures. Study participants were invited to comment on the logic of the conclusions as well as on the adequacy of their representations through the interpretations of the data.

### Limitations

While we worked hard to mitigate the gaps, a few limitations need to be taken into consideration. First, there has already been a mention of interviews being conducted online rather than in person, limiting the perception of physical, non-verbal cues that usually enrich the process of memoing. To mend this limitation, video conferencing was used which helped in creating conversational patterns. Next, we are aware of the timeliness of the data collection, and the fact that we have been examining a certain phenomenon while it was taking place. Arguably, we might obtain a different set of data should we conduct the research at a different moment in time, when interviewees have a retrospective view of events. Finally, as a minor note, we do acknowledge that English was used for all interviews, which oftentimes was not the native language. Nevertheless, due to the professional setting of the study in which English is widely used and accepted for everyday professional communication, we do not perceive this to have caused any impairment to the quality of data.

## Results and analysis

Our analysis brought up five code categories, namely (1) reflection on teaching, (2) reflection on students, (3) support and external factors, (4) online teaching and reactions, and (5) teacher emotions. In this categorisation, we made a distinction between (1) reflection on teaching, and (4) online teaching and reactions, especially since in the first category the coded segments indicated participants’ usual teaching approach, descriptions of learning through teaching experience, ways of creating learning conditions in pre-COVID times, and teaching ‘by feeling’ as a description of implicit pedagogical knowledge executed in the classroom. The codes grouped around online teaching contained reactions and adaptation to emergency online teaching, creating video recordings, comparing online and in-person experiences, remarks on ICT tools and skills, and seldom personal (home) situations and dilemmas.

**Fig. 1:**
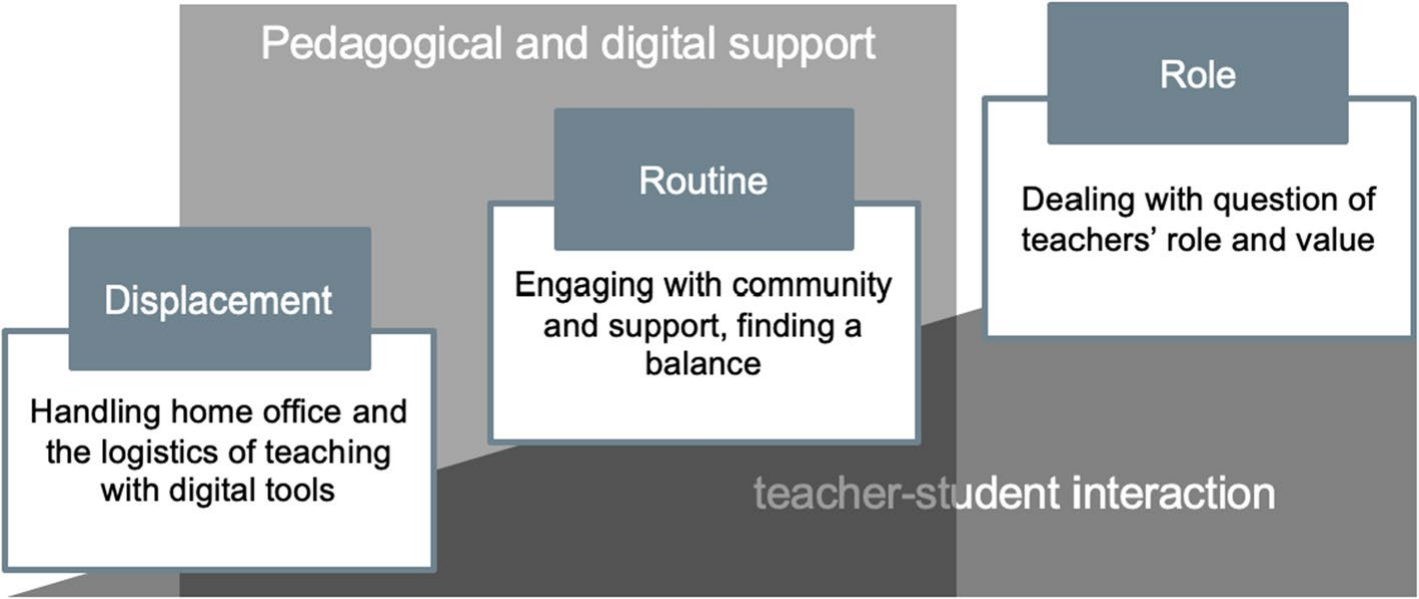
Visual representation of data analysis

In the following step of our analysis, we looked at the identified categories within a time perspective, particularly how events unfolded for the participants. In this way, we were able to develop four themes, of which three seemed to appear in a sequence and one underlying theme that ‘grows’ in its importance as the other themes emerge. Our attempt to visually represent this idea is depicted in Fig. 1.

Linearly with respect to the timeline of the lockdown, the first theme regarded the instant displacement, which carried with itself logistical adjustments to both working from home and working with technology. This theme encompassed codes combined from three categories, namely online teaching and reactions, support and external factors, and teacher emotions. In the visual model, however, pedagogical and digital support was added with a slight delay in respect to the timeline, as time was needed both for these services to quickly combine and provide support, as well as for teachers to realise where and how to seek for this support. The second theme that followed seemed less logistical, less oriented to technological functionality, and more concerned with pedagogical aspects of coping with the situation. The second category is related to the development of a routine that allows for comfortable teaching practice. Both of these categories, but in particular the second, were strongly reliant on community and knowledge sharing, as well as institutional pedagogical and digital support. This theme exposed codes from support and external factors, reflections on teaching, and online teaching and reactions.

At the creation of the third category, we noticed that once the perceived crisis of technological inadequacy and settling working conditions at home were fully or mostly resolved, the teachers seemed more perceptive of their role and what it means to teach in the new conditions. Thus, the third category carries deeper dilemmas related to teaching goals and pedagogical values, and as such stems from teachers’ reflections on teaching, and connects to teacher emotions as well as reflections on students. Furthermore, the component of reflection on students combined with some elements of reflections on teaching and teacher emotions provided a perspective of importance given to the teacher-student interaction. Hence, while the three previous themes seemed very distinctive in the analysis, we noticed the teacher-student interaction as a common thread binding them. As a leitmotif appearing at multiple points in the coded material and at several points in the timeline of the events, teacher-student interaction represented a common concern that, when placed in the conjunction with the three identified categories, had a gradual effect.

In the following, we will give further insights into each of the four themes, how they were captured in the interviews and how they may be described through the theoretical notions given in the previous parts of this paper.

### Displacement

While on rational level, teachers seemed to believe the lockdown was a reasonable reaction to the global situation, on the emotional level it did feel unprecedented.

My initial feeling was, it’s impossible that we will all be locked down. It was kind of surreal as I was still teaching on Wednesday (Dmitri, experienced teacher)

This speaks of the significance and extraordinary nature of the situation and almost all interviewed teachers, regardless of their teaching experience and form of teaching (e.g. large classes, labs), noticed that the emergency online teaching included different kinds of stress and fears. For many, there was something implicit concerning the sudden impersonality of their work, whether it was the discomfort of talking to the screen, the fear that students will not learn as much as they could, or just the fact that they are not in their normal classroom setting. Fiona, who teaches large first-year class and also has a student group in the laboratory, expresses this in her comment:

Honestly, I was scared about the fact that I was going to have to teach the classes online. Scared that I wouldn’t be up to the task, scared that I wouldn’t know how to do it, that students wouldn’t learn as much as they usually do, scared that it could be difficult to evaluate them in the exams (Fiona, mid-career teacher, email interview)

Like many others, Fiona did not have previous experience in teaching classes online; hence, her fears were related to her capacity to perform as well as she would in the classroom setting. Additionally, the fear of the teachers described through the interview except above also relates to student learning, particularly that the students’ knowledge acquisition will be crippled by being physically absent from the places of learning and evaluation.

For many teachers, the notion of teaching well at the beginning of the confinement related to being capable of fluently using the digital tools that they were confronted with. While there was a level of anxiety, even among the more experienced teachers, a notion of relief was noticeable when the interviewees realised that using Zoom for instance is not that difficult.

Zoom works well I must admit. It was easy to use for someone who’s reputed as not being fantastic with new technology (John, experienced teacher)

This kind of reassurance that digital tools can be tamed to establish reasonable functionality allowed for reflections on other aspects of teaching. For instance, there were comments on benefits to their learning, as well as opportunities to enhance a few methods used before the confinement:

I think for us it is good because we also have to learn how to use it. I mean, we are using computers every day, but there are a lot of options that we didn’t know before. It is also good for us to adapt (Laura, early-career teacher)

Furthermore, successful handling of the digital aspects of teaching under lockdown opened up a range of ideas about creating educational videos and their use in the new setting. For instance, ideas emerged as to what video lessons should contain, as well as how they can be adapted to a laboratory setting that is inherently bound to on-site equipment and experiential application.

I really think if students are going to watch something that’s recorded, it should be recorded as you record a MOOC. In other words, scenarize. You have to create a scenario (Gregor, experienced teacher)

I prepared these little videos. I use the [laboratory] setup and people can see me describing the setup and showing different aspects. And then I pause, and I start a new video on the whiteboard where I write equations or little schemes and then I pause again. They have to follow a document and reply to the questions. And, for example, it says look at Video 1 and the folder and then reply to this. I tried to do as much interaction as possible without interaction (Anne, early-career teacher)

In these excerpts, we notice that the initial fear of teaching with digital is somewhat replaced by an opportunity that digital can bring, and this was evident for teachers with little and for those with many years of experience. Gregor, who had previously prepared MOOCs, easily and quickly reflected that the educational videos should contain a sort of a scenario. A similar reflection emerged in an interview with Anne, who is a young teacher without previous online teaching experience, as she pointed out her strategy to engage students in laboratory classes which are dependent on discovery and hands-on learning.

Being in a situation to create digital educational artefacts has allowed teachers to reflect on the benefits of these artefacts. Not surprisingly, these benefits often revolved around their students, either from the perspective of supporting students who want to relisten some of the taught content or of using one video in different classes that follow the same content.

I had thought about that before, a long time before actually. Finally, when you think about it, you repeat every year the same type of course for different students. And ok, nothing replaces direct interaction and the atmosphere that you can have in the classroom where people not only ask questions to the professor, but they also interact with each other. It’s like when you go to a concert which you can watch over the TV. It’s not the same. But at the same time, recording it and having a formal reference of the course that students can listen to again when they prepare for the exams can help them understand better (Benjamin, experienced teacher in leadership position)

From these reflections, we could understand that teachers moved from basic technicalities to more elaborate ideas of online teaching. It is not surprising that the comfort of using the digital tools allowed for the development of a stabilizing routine, hence for an opening to handle more substantial issues.

### Developing a routine through experience and community

Among the interviewees, there were a few that were familiar with digital tools, and they noted that their previous experiences were helpful. The knowledge that was built before the lockdown increased the awareness of the institutional capacity to cope with the crisis. Additionally, some interviewees noticed that recurring experience in teaching a specific course helps in fine-tuning the content, understanding the balance of theory and practice, and how students learn the taught material.

I’ve been teaching this for now almost 4 years in a row with the same content and almost the same way of teaching, [and] you kind of know what is the knowledge they have and you can anticipate the questions. Every year they have almost the same questions. […] So, I tried to address as many questions as possible in my videos, trying to make it as clear as possible (Anne, early-career teacher, no prior online teaching)

Still, it is important to state that besides their own discoveries, the institution’s strong and capable support centres played a significant role in helping navigate through the unknown waters of the sudden campus closure. Throughout the lockdown, these centres provided solutions to an array of technical and pedagogical issues, and this was appreciated by the interviewees. Alongside the quick and adequate reaction from the support centres, in some cases, there was a notice of a horizontal peer-to-peer collaboration and knowledge sharing, as this passage points out:

In our section, in our small unit, it worked pretty well because we helped each other with the technical stuff and everybody started making these videos (Nikolai, mid-career teacher, with prior online teaching)

Even before the lockdown, Nikolai worked on creating video content and MOOCs for parts of his classes. As a passionate proponent of including videos as part of teaching materials for the first-year students, the chance of supporting other colleagues in his unit merged with an opportunity to create and use join materials that teachers can use among themselves.

While institutional and peer support helped build a relatively comfortable routine with some of the interviewees, there was still a notion of stress that the teachers pointed at, as John explains:

Talking to screen, I’m projecting the voice, and it is more tiring than normal. I mean, I find my throat worse than in normal condition. And, also the preparation and being ready to click the button. It is not the same as if you’re teaching at 3:15, then you are teaching at 3:15. This is a bit of a different stress (John, experienced teacher, no prior online teaching)

The awkwardness of teaching in front of the screen instead of in front of the live audience was noted by others too, and most of them needed to find a coping mechanism that would mediate the situation, like Jacques said: ‘It was about how to best cope with the situation and explain as best as we can in front of your computer rather than try to think that they [students] are there’ (Jacques, experienced teacher, no prior online teaching). Furthermore, some teachers experienced a drop in attendance by students for the synchronous online lectures and this created both frustrations and concerns. This was often compared with aspects of teaching that teachers valued in the pre-COVID situation, namely the needed interaction with students that helped them gather feedback, and make pedagogical decisions.

We did see from the interviewees that the concern about students and their learning was at the centre of their attention, even when all they could do is lecture through streaming or online recordings. Inevitably, without having much of the visual and spatial feedback that they rely on, the interviewees did reflect on what is their role in the new normal.

### Making meaning of teachers’ role

While most of the interviewees managed to find a relatively comfortable rhythm with the digital tools, the data shows that this was not entirely satisfying. For most teachers, using Zoom to record and transmit the knowledge was not the job nor the role they imagined as teachers. And while there was a noticeable acknowledgement of the benefits of recorded classes, the situation also brought in question making sense of the role of teachers in the newfound situation.

The plus side would be that now we have these classes and they are recorded. And next year we can do something different with them. But there’s also a very big question; if you have everything on a video, what do you do in the class with the students? And there you have to be innovative, I think (Dmitri, experienced teacher)

As Dmitri voiced out, rethinking what and how to structure classes is necessary especially if the main batch of knowledge and information is transmitted through the recorded lecture. The recorded lessons offer a one-directional activity that lacks student input and interaction. And through the interviews we have noticed expressions of emotions and concerns, as well as feelings of longing for the connection with the students, as these two excerpts show:

I honestly miss the interaction with the students. I appreciate the teaching part, especially because I have a small group. And you can really help them (Anne, early-career teacher)

I really miss the contact with the class. I don’t know what’s really going on with the whole class (Leopold, experienced teacher)

Both Anne and Leopold, two teachers that teach at different levels (i.e. labs and large classes), have reflected on the fact that without the student interaction, they feel they are not adequately informed on how students are doing in their learning. The lack of direct contact with students was a worrisome aspect of teaching under lockdown for all interviewed teachers. Most of them explained in great detail how the classes they teach are conceptually challenging and how they appreciate the moments when students ask questions or take advantage of breaks to seek further explanations. Also, teaching without students was described as ‘weird’ and ‘boring’:

About the feeling with the students, of course, it’s a very weird feeling right. Because suddenly you’re not talking anymore to the students (Dmitri, experienced teacher)

It’s a bit the connection that you don’t have any more with the students that can make it quite monotonous and repetitive and a bit boring (Jacques, mid-career teacher)

The choice of digital tools, which was primarily Zoom, had a significant impact on the reduced visual and verbal feedback between teachers and students. The videoconferencing platform could not provide the type of interaction that teachers needed, especially since the reaction to the new teaching model implied comparing it to the in-person teaching, as these two quotes capture:

I’ve been teaching for 28 years and I know what happens when I to the exercise rooms and I sit next to students. I see their scribbles. We talk about it, others are listening or not listening. There’re a million things happening. Live (Gregor, experienced teacher)

In the video you just repeat things the same way. What’s really needed after that is that you’re next to them and you can explain, you can phrase their questions and then you see what they don’t understand. And that’s where I have to step back and re-explain. The contact is really crucial (Dmitri, experienced teacher)

In these excerpts, we see how the role of teaching connects to the fact that teaching is highly tacit and based on situated judgements that stem from the interactions teachers have with their students. For teachers, tacit knowledge and appropriate pedagogical decisions have been situated in the physical context of the university, thus changing the social setting to a virtual and often asynchronous lecture feels disabling. In both Gregor’s and Dimitri’s reflections, teaching relies on the ability to correspond with students in a face-to-face, synchronous fashion.

In addition, more than their own conditions, teachers commented on the living conditions they imagined students to have and how living in small single rooms, potentially isolated from friends, peers, and campus life can be damaging for their feeling of motivation and overall happiness, both which play a role in learning. The concerns were particularly strong for teachers teaching first-year students, who oftentimes struggle with the volumes of subject matter in math and physics. For these teachers, as much as to some others, the lockdown brought more alertness of the large amount of content students need to cover and the suboptimal conditions in which the learning under lockdown takes place. Arguably, not having control over this made teachers truly question how they can fulfil their roles and make a purposeful impact on students and their learning.

## Discussion and conclusions

Taking into consideration the findings, we looked at the guiding research questions in an attempt to further explore the ways teachers experienced the abrupt change and what this change potentially means when looked at from the perspective of social practice.

The findings were encapsulated in a three-phased model tied with a thread of teacher-student interaction. This gradual model offers a point of departure for discussing how teachers’ experiences of change were interpreted, and it starts with the notion of being forced to exchange classrooms and laboratories for online, virtual spaces that are entered from their own homes. The technological aspect of this change, or knowing how to deal with digital tools and having a supporting institutional infrastructure, confirmed to be an important element in teachers’ experiences under the lockdown (Tartavulea et al., 2020; Damşa et al., 2021; Farnell et al., 2021), including for those who already had previous knowledge in using online platforms for teaching (Damşa et al., 2021). Even though the technological part came fairly easy for the teachers, moving to an emergency online mode did cause discomfort and a perceived loss of effectiveness (Bransford et al., 2007; Hammerness et al., 2007). This indicated that the hardships teachers experienced were not purely related to their digital competencies. Once teachers managed to reach a satisfying level of functionality with the digital tools, contentment with using the tools merely within the broadcast model remained rather low (Bourne et al., 2005). It can be debated that the selection of the ‘safer’ broadcasting telecommunication tools, such as Zoom, was a natural response to gain stability in adapting to the situation (Bransford et al., 2007).

Once the situation stabilised and the routine was established, there was a feeling of void. Unfulfilled expectations of student involvement and the fact that teaching was even less controllable than in the physical classroom setting (Hagenauer & Volet, 2014) came to the front and added to the anxiety and stress, making teachers experience negative emotions. For that given time, these negative emotions potentially impacted how teachers perceive online teaching and the possibility to progress and find solutions to their pedagogical dilemmas (Trigwell, 2012). To this analysis, it is also critical to bring in the context of engineering education being highly practical, where on-site and hands-on learning carries great importance. Conducting laboratory sessions or practical exercise sessions was seen as difficult, and participants struggled to recreate learning through inquiry by recording shorter sessions or pacing learning in cut-out videos. However, even the teachers that taught in large auditoriums noticed that the context of ‘taking to the screen’, not getting the visual feedback and not being able to ‘feel the room’ stripped their teaching practice from the one that they enjoy and feel satisfied with, the one in which they feel they can offer guidance, feedback or, as phrased by the teachers — where they can really help the students.

This said, displacement of teaching practice from the usual ‘spaces of knowledge’, as described by Haslanger (2018), lead to a necessity to question and potentially redefine the role of the teacher as an agent engaged in knowledge production with a responsibility towards knowledge distribution. From the data, we see that while teachers felt their responsibility to engage in the production and distribution of knowledge, the coordination and mode of participation in this practice has changed to a practice that potentially needed a different framework or mindset. By this, we point to the idea that the two modes, in-person and online teaching, do not rely on the same social practice, particularly as they do not engage the important aspects of the practice, like space, time and participation, in the same way. Therefore, comparing the two without taking into consideration the change in the social practice, might not lead to constructive developments in either.

In teachers’ reflections, there was a strong sense of loss of immediacy and the disruption of physical proximity (Andersen, 1979; Wubbels & Brekelmans, 2005) that allows for visual cues embedded in non-verbal communication and psychological connections through shared spaces (Walker & Gleaves, 2016). Lockdown disorientation in teaching came as a result of disrupting what was previously known as the shared meaning of the social practice of teaching (Haslanger, 2018; Mardahl-Hansen, 2019). The interaction, especially physical and visual, that happens in classrooms, labs, and auditoriums, as the spaces of learning and teaching, was nullified by the lockdown situation, and this left teachers seeking to reassert their purposive roles as teachers (D’eon et al., 2000). They could not rely on the situated pedagogical judgement based on their sense of the class as they would usually do, and it seemed difficult to find reasons to propose one or another activity without understanding how it might support creating a conducive learning environment.

Furthermore, the broken interaction with students impacted the meaning of teaching. In the campus setting, there was a shared notion of agency and space (Haslanger, 2018; Mardahl-Hansen, 2019), but when the patterns of actions got moved to private spaces without prior agreement or preparation, the previously mutually understood behaviour is lost. Teachers and students had to recreate a new set of norms by which they do education, and without other actors that play a role in the social construct of education on campus, this impacted the fact that teachers became central to student learning. When on campus, students have a setting in which they can learn in different spaces and from different actors, such as their peers, coaches, librarians, and through activities in student associations, informal meals and breaks, etc. The dimensions of social theory of learning (Wenger, 2009) that are normally situated in multiple interactions, got limited to online interactions with teachers’ lessons, and sporadic contacts through online social networks. Being reduced to their own private setting and exposed only to the recorded or live-streamed lectures arguably created more pressure on learning through the online lecture driven by one actor — the teacher. The fact that the switch to online distant education happened abruptly meant also that teachers were not prepared to utilise the teaching presence properly which was described by Garrison et al. (2003) would support designing and facilitating social and cognitive processes in developing meaningful learning outcomes.

While this research did not target student experiences, teachers’ reflections on student suboptimal learning conditions meant that they, the teachers, feel that on-site learning is a more favourable form for students. Beyond teachers’ understanding of student learning as more holistic when done through multiple interactions, it is also possible to argue that the teachers’ perspective of on-campus learning is limited to their feeling of control and situatedness of their teaching (Hagenauer & Volet, 2014). In other words, ‘feeling the room’ and knowing when to react in one’s teaching talks in both ways, as (a) teaching being a highly complex and interactive verbal and non-verbal communication process and (b) misconception that students learning is a result of teacher’s teaching (Mardahl-Hansen, 2019). These two explanations can very well exist at the same time, and both need to be questioned in a social practice that reduces the physical, synchronous contact.

The sense of situatedness and the value of materiality is particularly evident in engineering education; however, beyond that, looking at the socially engaged roles teachers and other actors have in constructing meaning in education is difficult and necessary to question and develop when moving to a different social practice, such as online education. From the interviews, we see references to a teaching practice that is reliant on a well-established, internalised social agreement that cannot be copied in an online setting. Hence, reflecting on how teaching can be re-constructed should be a process that is taken with great care, especially if the aim is to achieve the higher-order learning outcomes through student–teacher communities (Garrison et al., 2003). The results of this study indicate that most of the teachers’ reflections were reactionary which can be easily argued as adequate given that the timeline of the study was the first wave of COVID-related lockdowns. Nevertheless, while this crisis could still bring an opening to examining social practice of teaching and learning, it is reasonable to post a question on when and how do teachers reflect on their teaching, especially on the aspects of change and development in social practice?

In conclusion, while there is probably no right way to prepare for a crisis like a global pandemic, we can draw several conclusions that can be useful for uncertain future situations. It is essential to appropriate pedagogical tools, online and in-person, to support an interactive, constructivist relationship with students, mainly for teachers to truly tap into their capacity to bring the most adequate judgements on creating conditions for learning. Furthermore, teachers who are pedagogical polyglots are also less likely to experience anxiety with sudden and unexpected changes. However, even though tools and methods are essential for teachers’ self-confidence, we do maintain that change in teaching practice goes beyond a single tool or competence. Equipping teachers with an array of tools, methods, and approaches helps in preparing them for the uncertainty, but the instances of professional development need to be well tied with what they see as the purpose of teaching and in line with their reasoning around what creates the most optimal learning environment. Additionally, a widespread change in teaching practice needs to be negotiated among teachers, and potentially include other actors such as students and leadership, since the norms that govern teaching practice are communal, and created and enacted through communities rather than individuals.
